# An epigenetic DNA methylation clock for age estimates in Indo‐Pacific bottlenose dolphins (*Tursiops aduncus*)

**DOI:** 10.1111/eva.13516

**Published:** 2022-12-15

**Authors:** Katharina J. Peters, Livia Gerber, Luca Scheu, Riccardo Cicciarella, Joseph A. Zoller, Zhe Fei, Steve Horvath, Simon J. Allen, Stephanie L. King, Richard C. Connor, Lee Ann Rollins, Michael Krützen

**Affiliations:** ^1^ Evolutionary Genetics Group, Department of Anthropology University of Zurich Zurich Switzerland; ^2^ School of Earth and Environment University of Canterbury Christchurch New Zealand; ^3^ Cetacean Ecology Research Group, School of Natural Sciences Massey University Auckland New Zealand; ^4^ Global Ecology, College of Science and Engineering Flinders University Adelaide, South Australia Australia; ^5^ Evolution & Ecology Research Centre, School of Biological, Earth and Environmental Sciences University of New South Wales Sydney, New South Wales Australia; ^6^ Department of Biostatistics, Fielding School of Public Health University of California Los Angeles Los Angeles, California USA; ^7^ Department of Statistics University of California Riverside, California USA; ^8^ Department of Human Genetics, David Geffen School of Medicine University of California Los Angeles Los Angeles, California USA; ^9^ Altos Labs, San Diego Institute of Science San Diego, California USA; ^10^ School of Biological Sciences University of Bristol Bristol UK; ^11^ School of Biological Sciences University of Western Australia Crawley, Western Australia Australia; ^12^ Biology Department UMASS Dartmouth North Dartmouth, Massachusetts USA

**Keywords:** aging, bottlenose dolphin, epigenetic clock

## Abstract

Knowledge of an animal's chronological age is crucial for understanding and predicting population demographics, survival and reproduction, but accurate age determination for many wild animals remains challenging. Previous methods to estimate age require invasive procedures, such as tooth extraction to analyse growth layers, which are difficult to carry out with large, mobile animals such as cetaceans. However, recent advances in epigenetic methods have opened new avenues for precise age determination. These ‘epigenetic clocks’ present a less invasive alternative and can provide age estimates with unprecedented accuracy. Here, we present a species‐specific epigenetic clock based on skin tissue samples for a population of Indo‐Pacific bottlenose dolphins (*Tursiops aduncus*) in Shark Bay, Western Australia. We measured methylation levels at 37,492 cytosine‐guanine sites (CpG sites) in 165 samples using the mammalian methylation array. Chronological age estimates with an accuracy of ±1 year were available for 68 animals as part of a long‐term behavioral study of this population. Using these samples with known age, we built an elastic net model with Leave‐One‐Out‐Cross‐Validation, which retained 43 CpG sites, providing an *r* = 0.86 and median absolute age error (MAE) = 2.1 years (5% of maximum age). This model was more accurate for our data than the previously published methylation clock based on skin samples of common bottlenose dolphins (*T. truncatus*: *r* = 0.83, MAE = 2.2) and the multi‐species odontocete methylation clock (*r* = 0.68, MAE = 6.8), highlighting that species‐specific clocks can have superior performance over those of multi‐species assemblages. We further developed an epigenetic sex estimator, predicting sex with 100% accuracy. As age and sex are critical parameters for the study of animal populations, this clock and sex estimator will provide a useful tool for extracting life history information from skin samples rather than long‐term observational data for free‐ranging Indo‐Pacific bottlenose dolphins worldwide.

## 1 INTRODUCTION

Accurate determination of an animal's age is a central criterion for understanding many aspects of its life, such as growth rate, age at maturity, peak reproductive performance, and life span; making age a crucial parameter to unravel key life history characteristics (Stearns, 1976). Furthermore, at the population level, large‐scale demographic age and generation time information are critical for assessing population viability (Heydenrych et al., 2021; Manlik et al., 2022). However, estimating age in free‐ranging animals is often difficult, particularly for long‐lived, highly mobile species, such as cetaceans (whales, dolphins, and porpoises; Read et al., 2018). While age information can be reliably gained from behavioral records, this requires a long‐term study effort, typically spanning multiple decades, until the entire age range of individuals in the population can be determined.

Alternatively, age can be estimated via the correlation of certain morphological or molecular traits with the known age of individuals. In mammals, most morphological age estimators suffer from low accuracy or require highly invasive procedures, rendering them unfeasible for application to many individuals. Toothed whales (odontocetes), for example, are often aged approximately via their total body length, which allows only a crude estimation of life‐stage (i.e., calf/juvenile/adult; Betty et al., 2022; Chivers, 2009). Another, more accurate method of aging odontocetes is via longitudinal sectioning and quantification of growth layers in their teeth. While this method is deemed relatively robust, differing levels of tooth wear depending on age and feeding techniques can confound results (Hohn & Fernandez, 1999; Perrin et al., 1980; Waugh et al., 2018). Furthermore, this method is largely restricted to deceased individuals due to its invasiveness and is, thus, logistically unfeasible for the majority of free‐ranging odontocetes (but see Hohn et al., 1989). In some species, the Indo‐Pacific bottlenose dolphin (*Tursiops aduncus*) for example, morphological traits such as skin speckling patterns can indicate age, but the approach is limited by a number of factors, including the observers' ability to image the entire body, variation between individuals in speckling rates, and differences in ‘capture’ probability across a population, and thus results are approximate at best (Krzyszczyk & Mann, 2012; Yagi et al., 2022). The idea of using telomere length as an indicator of age has received much attention in recent decades, but the decline in telomere length is weak and too variable across vertebrate classes to deliver reliable estimates (Dunshea et al., 2011; Jylhävä et al., 2017; Olsen et al., 2012).

Recent advances in sequencing technology have opened new avenues for precise age determination using DNA methylation data. The resultant ‘epigenetic clocks’ are based on correlates between age and DNA methylation of specific cytosine‐guanine (CpG) sites and are currently considered the best age predictors available (De Paoli‐Iseppi et al., 2017; Guevara & Lawler, 2018; Jylhävä et al., 2017). The CpG sites used to build epigenetic clocks can also be used to reliably predict sex. This is particularly useful for species that are difficult to observe and lack clear sexual dimorphism, such as some odontocetes. Robeck, Fei, Lu, et al. (2021) built a multi‐species clock to estimate age and predict sex for odontocetes, which was cross‐validated for nine species (common bottlenose dolphin *T. truncatus*, beluga *Delphinapterus leucas*, killer whale *Orcinus orca*, Pacific white‐sided dolphin *Lagenorhynchus obliquidens*, short‐finned pilot whales *Globicephala macrorhynchus*, rough‐toothed dolphin *Steno bredanensis*, Commerson's dolphin *Cephalorhynchus commersonii*, common dolphin *Delphinus delphis*, harbour porpoise *Phocoena phocoena*). Multi‐species clocks effectively estimate age and sex of individual members of various species simultaneously and, thus, facilitate conservation efforts by enabling the investigation of population viability of multiple species with a single tool (Robeck, Fei, Lu, et al., 2021). Nevertheless, accuracy of age predictions can be improved with species‐specific clocks (Field et al., 2018; Zhang et al., 2019), several of which currently exist for odontocetes, including belugas (Bors et al., 2021) and common bottlenose dolphins (Barratclough et al., 2021; Beal et al., 2019; Robeck, Fei, Haghani, et al., 2021).

The Shark Bay Indo‐Pacific bottlenose dolphin population off Monkey Mia, Western Australia, has been studied in considerable depth since the early 1980s (Connor & Smolker, 1985), making it one of the best‐known dolphin populations in the world (Allen et al., 2016; Connor & Krützen, 2015; Krützen et al., 2004). Photo‐identification records and the social behavior of Shark Bay's dolphins have been documented for 40 years, so year of birth and sex are known for most individuals (Connor & Krützen, 2015; King et al., 2021). To date, when no birthdate is known, the age of dolphins could only be roughly estimated using a suite of approximate measures, such as birth of first calf (females), first herding of females for reproductive purposes (males), individual sighting histories, and skin speckling patterns (Krzyszczyk & Mann, 2012). The lack of reliable age estimates has been a limiting factor in research relating to dolphin reproduction and life history (but see Karniski et al., 2018; Taylor et al., 2007). Outside Shark Bay, Indo‐Pacific bottlenose dolphins are widely distributed, ranging from coastal areas in the Indian Ocean throughout Southeast Asia to parts of the western Pacific (Wang, 2018). The development of epigenetic aging clocks requires samples from animals with known ages for calibration, so the Shark Bay population offers an exceptional opportunity to build a species‐specific clock for Indo‐Pacific bottlenose dolphins using samples collected from wild animals.

Here, we present a species‐specific age estimation clock as well as sex predictor based on DNA methylation data extracted from skin samples of Shark Bay's Indo‐Pacific bottlenose dolphin population. We further compare the accuracy of age estimates from this clock for our data with that of the multi‐species odontocete clock (Robeck, Fei, Lu, et al., 2021) and the common bottlenose dolphin clock (Robeck, Fei, Haghani, et al., 2021). Our epigenetic clock will inform species conservation management, as well as studies focusing on population biology, social organisation and behavior, throughout their broad range.

## 2 METHODS

### 2.1 Study site and sample collection

Skin samples and birthdate estimates of individual dolphins included in this study are part of a long‐term research project in Shark Bay, Western Australia. Shark Bay is a large, semi‐enclosed, subtropical embayment along the central coast of Western Australia. Here, we used skin samples from 168 free‐ranging Indo‐Pacific bottlenose dolphins collected between 1995 and 2019 using a specialised biopsy system for small cetaceans (Krützen et al., 2002). Age was known from a long‐term photo‐identification study (Connor & Krützen, 2015), with an accuracy of at most ±1 year for 68 animals and ±2 years for 84 individuals (including the 68 with ±1 year). For the remaining 82 samples, age was known with an accuracy of >2 years. Skin was stored in RNAlater (Thermo Fischer Scientific) or saturated NaCl and 20% dimethyl‐sulfoxide (DMSO) solution at −20°C in the field and −80°C in the laboratory. We extracted genomic DNA from skin samples using a Quick‐DNA™ Miniprep Plus Kit (Zymo) and subsequently purified the DNA with a DNA Clean & Concentrator Kit (Zymo) following the manufacturer's instructions. We measured DNA concentration using a QUBIT 4 fluorometer (Thermo Fisher Scientific).

### 2.2 DNA methylation data

We used a custom Infinium methylation array (HorvathMammalMethylChip40) assembled with 37,492 CpG sites to profile DNA methylation arrays (as described in Arneson et al., 2022). We used unsupervised hierarchical clustering analysis based on the inter‐array correlation to visually detect technical outliers which we then removed from further analysis.

### 2.3 Molecular sex identification

We determined sex by multiplexed polymerase chain reaction amplification of the two sex chromosome‐specific loci ZFX and SRY using the primers P1‐5EZ, P2‐3EZ (Aasen & Medrano, 1990) and Y53‐3C, Y53‐3D (Gilson et al., 1998), respectively. We used gel electrophoresis in combination with stained DNA bands (GelRedTM) and UV light (E‐Box; Vilber) to visually determine sex based on the number of visible bands.

### 2.4 Elastic net regression to predict age and sex

We built two epigenetic clocks: one using 68 samples with an age accuracy of at most ±1 year, and one using a total of 84 samples: the 68 samples with ±1 year accuracy plus an additional 16 samples with an age accuracy of at most ±2 years. The remaining 82 samples were not used to build the clocks as, for these, age was known with an accuracy of >2 years. They were, however, included in the sex predictor (see below). To build these clocks, we used an elastic net model using the R package glmnet (Friedman et al., 2010, 2022; R Core Team, 2022). Elastic net models are suitable in cases where the number of predictor variables exceeds the number of observations and where some of the predictor variables are correlated, as is often the case for genomic data. We set the elastic net mixing parameter alpha to 0.5 to equally shrink (alpha = 0) and remove (alpha = 1) predictors, as is customarily done in epigenetic clocks (Lu et al., 2021). We used the function cv.glmnet to automatically determine the lambda penalty parameter via n‐fold internal cross‐validation (*n* being the numbers of samples included). Using this lambda, we then used a leave‐one‐out‐cross‐validation (LOOCV) approach to estimate the age of individual dolphins. For each sample in the dataset, the LOOCV omits one sample, then fits the clock on the remaining data, thereby predicting the age of the omitted sample (Zhang, 1993; Zou & Hastie, 2005). For the clock based on 84 samples, we weighted each sample within the glmnet function based on the accuracy of its age (±1 = 1.0, ±2 years = 0.5). We assessed the accuracy of our epigenetic clocks using the age correlation *r* (the Pearson correlation between the predicted epigenetic age and the age determined through long‐term observations), and the median absolute error (MAE) between the predicted and the observed age. To account for the accelerated aging process before reaching sexual maturity (Lu et al., 2021), we used a log‐linear transformation based on the average age of sexual maturity (7 years, Kemper et al., 2019), as suggested by (Horvath, 2013). Individuals from Shark Bay seem to become sexually mature at ~10–12 years of age, based on observational and demographic data including the birth of the first calf for females and first herding of females for reproductive purposes for males (Karniski et al. (2018); Shark Bay Dolphin Research, unpublished data). Kemper et al. (2019), on the other hand, investigated the number of corpora in ovaries and the number of spermatozoa and stage of spermatogenesis to infer age of sexual maturity. Since such data do not exist for Shark Bay individuals, we used estimates provided by Kemper et al. (2019).

To assess our clock's performance in relation to that of other epigenetic clocks, we subsequently compared our species‐specific epigenetic clocks with the previously published species‐specific common bottlenose dolphin clock (Robeck, Fei, Haghani, et al., 2021) and the multi‐species Odontocete Epigenetic Aging Clock (OEAC; Robeck, Fei, Lu, et al., 2021) for skin tissues as well as for skin and blood. For this, we applied these four clocks to our samples of known age and compared their respective *r* values with that of our species‐specific clocks for Indo‐Pacific bottlenose dolphins.

To predict sex for each sample, we used a similar approach to that outlined above, with the difference that we ran a binomial elastic net regression, encoding sex as a binary outcome variable (0 = female, 1 = male), and did not use an LOOCV approach. We used 165 samples (three technical outliers were removed from the total dataset of 168, see results) as we had sex information for the whole dataset, and sample sizes for males (92) and females (73) were reasonably balanced. If the predicted probability was >0.5, the sample was considered male.

## 3 RESULTS

We profiled DNA methylation patterns from 168 tissue samples of dolphins (92 males, 76 females), 68 of which had known ages with an accuracy of ±1 year and were aged between 0 and 37 years (mean ± SD = 14.6 ± 8.0, 43 males, 25 females). The unsupervised hierarchical clustering analysis identified three technical outliers, which were removed from further analysis (for details, see Figure S1).

### 3.1 Epigenetic aging model

**FIGURE 1:**
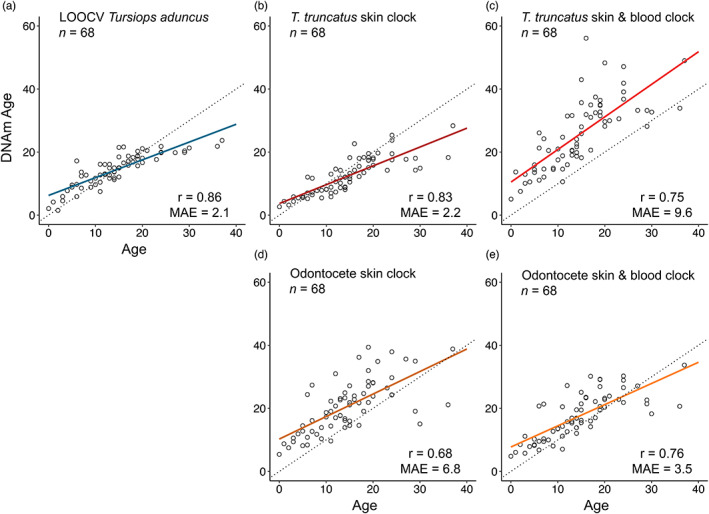
Epigenetic ages calculated using elastic net regression models with leave‐one‐out‐cross‐validation applied to 68 skin samples of Indo‐Pacific bottlenose dolphins (*Tursiops aduncus*) for which age was known with an accuracy of ±1 year. Ages were calculated using (a) the species‐specific clock developed in this study, (b) the skin clock developed for *Tursiops truncatus* (Robeck, Fei, Haghani, et al., 2021), (c) the skin and blood clock developed for *T. truncatus* (Robeck, Fei, Haghani, et al., 2021), (d) the skin clock for multi‐species odontocetes (Robeck, Fei, Lu, et al., 2021), and (e) the skin and blood clock for multi‐species odontocetes (Robeck, Fei, Lu, et al., 2021). Regression lines are shown in blue (*T. aduncus* clock), orange (*T. truncatus* clocks) and red (multi‐species odontocete clocks), dotted diagonal indicates a perfect correlation (*y* = *x*), and points represent individual animals. Pearson correlation (*r*) and median absolute error are given for each model.

The elastic net model with LOOCV produced accurate age predictions, with a correlation between the epigenetic age and the known age from observational data of *r* = 0.86, and a MAE = 2.1 years (Figure 1). The model showed a slight trend of overestimating the age of younger animals and underestimating the age of older animals (regression slope = −4.407; regression y‐intercept = 1.312). The final version of the clock based on all 68 samples where age was known with an accuracy of at most ±1 year retained 43 CpG sites (Table S1). The results of our second clock, based on a larger number of individuals (*N* = 84) but whose age estimates are less accurate (±2 years), were in line with the first clock, albeit slightly less accurate. Details of this clock can be found in the Supporting Information.

The previously published species‐specific clock for common bottlenose dolphins (Robeck, Fei, Haghani, et al., 2021) based on skin samples, and the clock based on skin and blood samples, predicted age for our samples with an accuracy of *r* = 0.83 and *r* = 0.75, and MAE = 0.22 and MAE = 9.6, respectively. The multi‐species odontocete clock for skin samples and for skin and blood samples (Robeck, Fei, Lu, et al., 2021) predicted age for our samples with an accuracy of *r* = 0.68 and *r* = 0.76, and MAE = 6.8 and MAE = 3.5 years, respectively (Figure 1).

### 3.2 Epigenetic sex predictor

The model estimating sex based on methylation patters of 165 samples retained 179 CpG sites. Using a threshold of 0.5 (samples >0.5 were considered male), the model predicted sex with 100% accuracy and did not misidentify any samples. For males, probabilities of being categorised as male ranged from 0.994 to 0.996. Female probabilities to be assigned to the male category were low, ranging from 0.004 to 0.009.

## 4 DISCUSSION

This study presents a robust and accurate (*r* = 0.86, MAE = 2.1) epigenetic clock for the aging of Indo‐Pacific bottlenose dolphins based on skin tissue. To date, five other epigenetic clocks exist for odontocetes, using skin and/or blood samples: three for common bottlenose dolphins (Barratclough et al., 2021; Beal et al., 2019; Robeck, Fei, Haghani, et al., 2021), one for belugas (Bors et al., 2021), and one multi‐species odontocete clock, which was cross‐validated with nine species (Robeck, Fei, Lu, et al., 2021). While Beal et al. (2019) used a pyrosequencing approach based on 13 CpG sites correlated with age, the remaining clocks were built using the HorvathMammalMethylChip40 with 37,492 CpG sites, as we did here. It is possible to cross the species boundary with the same Infinium chip (Illumina), due to the special array platform, focusing on ultra‐conserved cytosines (as described in Arneson et al., 2022). Clocks for skin samples ranged in precision from *r* = 0.74 (Beal et al., 2019; Bors et al., 2021), to 0.92 (Robeck, Fei, Lu, et al., 2021), to 0.95 (Robeck, Fei, Haghani, et al., 2021).

Considering the longevity of the species, the clock that was developed for the common bottlenose dolphin (*T. truncatus*), a sister species to the Indo‐Pacific bottlenose dolphin (*T. aduncus*), performed extremely well for our samples and returned an MAE of 2.2, very close to the MAE of 2.1 for our *T. aduncus* clock and lower than the MAE returned by the multi‐species odontocete clock (MAE = 6.8). While this result highlights the benefit of increased accuracy of a species‐specific clock where possible, it also shows that age estimates derived from a clock developed for a sister or otherwise closely related species can be very similar. The recommended sample size of 70 to build a clock (Mayne et al., 2021) may be impossible to achieve for rare, elusive or otherwise difficult‐to‐sample species. Similarly, analytical costs for such a high quantity of samples might prevent the development of a species‐specific clock in some cases. In those situations, using an available clock based on sister species, as well as a multi‐species clock, can prove useful.

It is common for epigenetic clocks to slightly overestimate the age of younger individuals and underestimate the age of older animals (Beal et al., 2019; Bors et al., 2021; Polanowski et al., 2014), a pattern apparent in the clock presented here. While we endeavored to include samples with ages covering the complete lifespan of free‐ranging Indo‐Pacific bottlenose dolphins, collecting skin samples from individuals under 2 years of age is avoided and individuals at the end of a species' maximum lifespan are scarce outside of captivity. As a result, our dataset includes only six animals each below 5 and above 25 years of age. The clock could be further improved by including more individuals at the extremes of their age distribution, particularly older ones (Mayne et al., 2021). The slightly less accurate result of the clock developed with 84 samples, 16 of which had known ages with an accuracy of ±2 years (Figure S1), highlights the need for accurate ages to train the clock. With a larger sample size of accurate ages (±1 year or less), our clock might be further enhanced. However, even epigenetic clocks trained on many samples across all age groups tend to show some variation, which is thought to occur due to individuals aging at different rates (Jylhävä et al., 2017). Individuals that are estimated to be older than their true age are thus thought to age faster. The rate of aging can be influenced by various biotic and abiotic variables, such as number of offspring (Shirazi et al., 2020), early life adversity (Colich et al., 2020), diet (Fitzgerald et al., 2021; Weindruch et al., 2001), and environmental pollutants (Liu et al., 2021).

Methylation rates are highly conserved in blood. Thus, clocks based on blood samples tend to be slightly more precise and accurate (Robeck, Fei, Haghani, et al., 2021; Robeck, Fei, Lu, et al., 2021). This was reflected in our results when applying the multi‐species odontocete clocks to our data, as age predictions improved when using the skin and blood clock versus the skin clock (MAE = 3.5 and MAE = 6.8, respectively, Figure 1). However, this was not the case for the *T. truncatus* clock (Figure 1). Here, the skin and blood clock performed worst of all clocks tested for our samples, greatly overestimating ages across almost all samples. As our samples stem from skin biopsies and we do not have access to blood samples, we were unable to test if the *T. truncatus* skin and blood clock performs better on blood samples from *T. aduncus*. It is possible there are more differences between the blood epigenome of *T. truncatus* and *T. aduncus* than between their skin epigenome, which would explain why the skin and blood clock performs worse than the skin clock for our data.

While it can be of value to use blood samples for the development and/or application of epigenetic clocks, the difficulty of collecting blood versus skin from free‐ranging odontocetes restricts blood sampling almost exclusively to captive individuals. Despite being based exclusively on skin samples, the epigenetic clock presented here estimated reliable ages for Indo‐Pacific bottlenose dolphins.

In addition to estimating the age of individuals, we also used the methylation data to predict sex, because methylation rates on CpG sites can be sex‐associated (Robeck, Fei, Lu, et al., 2021). Our sex predictor was accurate across all 165 individuals and can thus be reliably used to determine the sex of Indo‐Pacific bottlenose dolphins. Epigenetic sex estimators have also proven highly accurate in other odontocetes (Bors et al., 2021; Robeck, Fei, Haghani, et al., 2021; Robeck, Fei, Lu, et al., 2021). The CpG sites retained in the model are likely associated with sex chromosomes (Bors et al., 2021; Robeck, Fei, Haghani, et al., 2021).

The epigenetic clock presented here predicts age and sex accurately, thereby providing a reliable tool to estimate age and sex for wild Indo‐Pacific bottlenose dolphin populations. This clock was built exclusively using samples collected from one population in Shark Bay, Western Australia. It would be of interest to compare its performance in this population with samples from other populations throughout the range of Indo‐Pacific bottlenose dolphins. Given the close result obtained with the common bottlenose dolphin clock, it is likely that the clock presented here will also estimate reliable ages for other populations of Indo‐Pacific bottlenose dolphins. Age is a crucial parameter in population biology and conservation, so this clock represents a useful tool for ongoing biological research.

## CONFLICT OF INTEREST

SH is a founder of the non‐profit Epigenetic Clock Development Foundation, which plans to license several of his patents from his employer UC Regents. The other authors declare no conflicts of interest.

## Supporting information


Figure S1

## Data Availability

Data will be archived either as part of the data release from the Mammalian Methylation Consortium or via GEO (Gene Expression Omnibus, https://www.ncbi.nlm.nih.gov/geo/). Code will be made available on https://github.com/kjopeters/Epigenetic‐clock‐Tursiops‐aduncus. The mammalian methylation array is available from the non‐profit Epigenetic Clock Development Foundation.
