# Supplementary material for: An epigenetic DNA methylation clock for age estimates in Indo‐Pacific bottlenose dolphins (Tursiops aduncus)
Source: Evol Appl. 2022 Dec 15;16(1):126–33. doi: 10.1111/eva.13516 (PMC9850008; doi:10.1111/eva.13516)
Supplement: Supplementary file 1 — Figure S1 [file EVA-16-126-s001.docx]

SUPPLEMENTARY MATERIAL

**An epigenetic DNA methylation clock for age estimates in Indo-Pacific bottlenose dolphins (*Tursiops* *aduncus*)**

**Results for second clock (*n* = 84)**

The elastic net model with Leave-One-Out-Cross-Validation (LOOCV) produced accurate age predictions, with a correlation between the epigenetic age and the observed age of r = 0.84, and a median absolute error of MAE = 2.3 years (Figure S1). Like the first clock, the model showed a slight trend of overestimating the age of younger animals and underestimating the age of older animals (regression slope = -4.883; regression y-intercept = 1.347). The final version of this clock, based on all 84 samples, retained 40 CpG sites (Table S1).

**Table S1** CpG sites retained by the final elastic net regression model to estimate epigenetic age from a) 68 and b) 84 skin samples of Indo-Pacific bottlenose dolphins (*Tursiops aduncus*) and their associated model coefficients.

| **a)** | | **b)** | |
| --- | --- | --- | --- |
| **Probe ID** | **Model coefficient** | **Probe ID** | **Model coefficient** |
| (Intercept) | 8.26626096 | (Intercept) | 9.48987947 |
| cg00422680 | -0.005324 | cg00234132 | -1.26326 |
| cg00585163 | 4.96830767 | cg00906973 | 3.68580864 |
| cg02317025 | 2.86108583 | cg02317025 | 2.77656331 |
| cg04453471 | -1.7218669 | cg02334175 | -0.2967102 |
| cg05314634 | 0.78200861 | cg05366673 | -0.1996498 |
| cg05366673 | -0.2848903 | cg06472065 | -0.6803232 |
| cg06472065 | -0.4143149 | cg07383477 | -0.1869027 |
| cg07019638 | 0.51849018 | cg08152447 | 1.14775467 |
| cg07383477 | -0.1136606 | cg08171000 | 0.86071207 |
| cg08152447 | 0.88324542 | cg08622677 | 1.27464649 |
| cg08538581 | -2.5577752 | cg09227056 | 1.96180699 |
| cg08622677 | 1.55700878 | cg09753325 | -1.2673797 |
| cg09227056 | 2.8043246 | cg10235290 | -0.6454717 |
| cg09363187 | 0.5295032 | cg10561550 | -0.1783722 |
| cg09753325 | -1.3068578 | cg11260459 | -0.4930064 |
| cg12435096 | -5.7766273 | cg12435096 | -2.0117706 |
| cg12451099 | 0.43851243 | cg12562732 | 5.15772087 |
| cg12562732 | 1.34560402 | cg12981876 | -0.6962553 |
| cg12981876 | -0.5560994 | cg13237109 | -0.4138139 |
| cg13237109 | -0.1694485 | cg13698738 | -0.1618021 |
| cg13808339 | 0.49407698 | cg13808339 | 5.3894023 |
| cg14427009 | 1.21970944 | cg14127056 | 0.11133562 |
| cg15976167 | -0.3531412 | cg14667871 | 5.66414783 |
| cg16524928 | 5.58939248 | cg15659936 | 0.72035118 |
| cg16606638 | 0.33856994 | cg15976167 | -0.3843558 |
| cg16947316 | -0.2238861 | cg16606638 | 0.57670117 |
| cg17257156 | 3.38060225 | cg17183991 | 0.18157631 |
| cg18468088 | 0.41410491 | cg18753125 | 0.02101487 |
| cg18814344 | -0.1568011 | cg18980531 | -5.7957683 |
| cg19529849 | 1.45329749 | cg19523029 | -0.0649796 |
| cg19873240 | 1.01394613 | cg19873240 | 0.35840884 |
| cg20421508 | 0.39024088 | cg21691694 | 26.96612 |
| cg21056089 | -1.5440407 | cg22306928 | 0.99277122 |
| cg21671607 | -1.8502058 | cg22712872 | -0.7321486 |
| cg21691694 | 31.8819289 | cg23360751 | -0.4160723 |
| cg22306928 | 8.38090461 | cg23576695 | -1.8280034 |
| cg23424255 | -0.0471634 | cg25354602 | 1.25937145 |
| cg23576695 | -2.4199807 | cg25408314 | -1.7891587 |
| cg24395509 | -1.752412 | cg26150462 | 0.39665927 |
| cg24826280 | -0.1176611 |  |  |
| cg25339883 | 1.33037132 |  |  |
| cg25402449 | -0.3050687 |  |  |
| cg25943999 | -0.2496476 |  |  |

# Figure S1 Unsupervised hierarchical clustering of *T. aduncus* skin samples. “Branch” reflects the respective cluster, “Age” encodes observed age (red is a high age, white low age), “Sex” encodes sex (pink = female, blue = male). The low height values (y-axis) indicate, overall, high inter-array correlations and high quality. The samples from the grey clusters (N = 3) were excluded from the analyses.


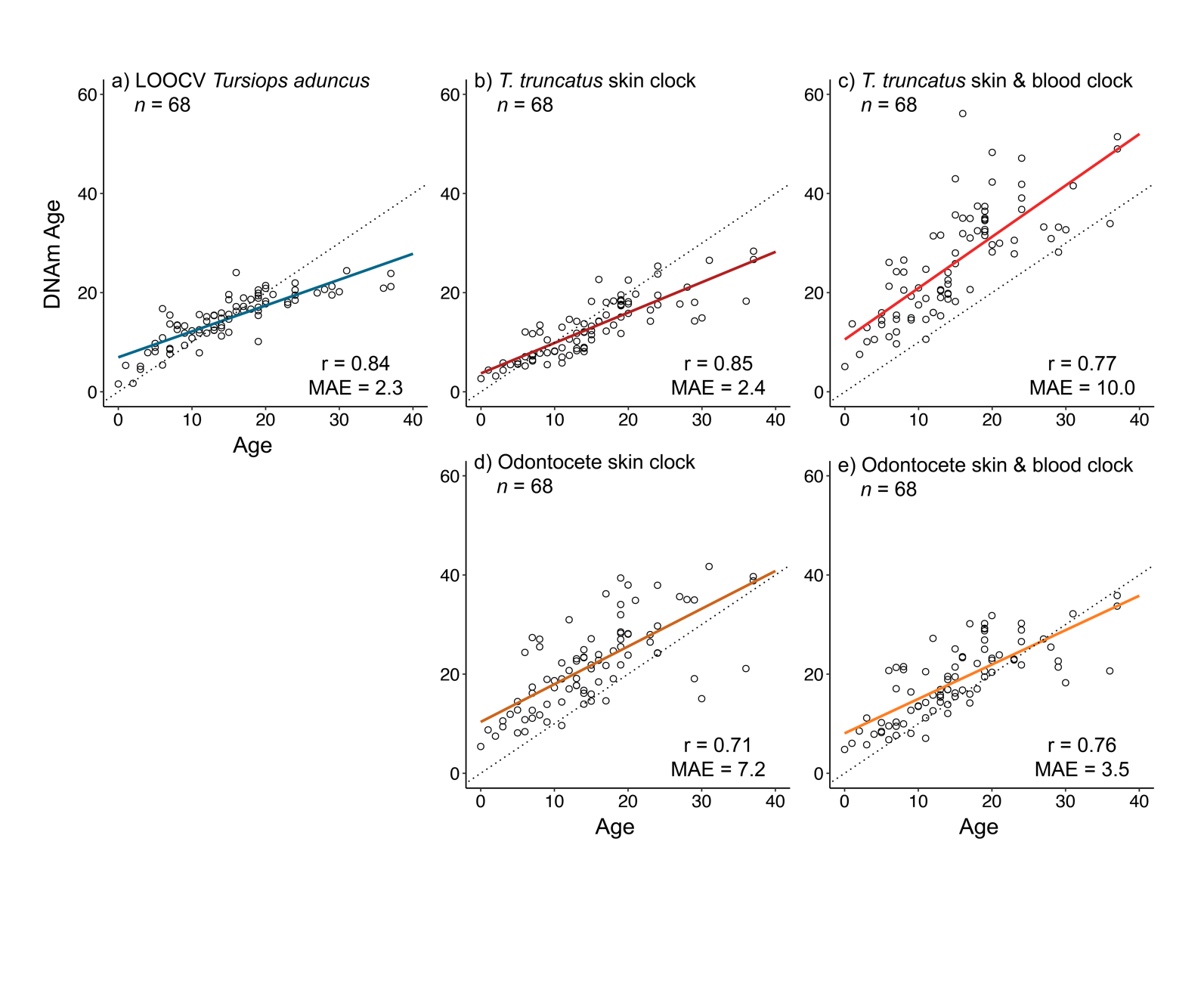
**Figure S2** Epigenetic ages calculated using elastic net regression models with Leave-One-Out-Cross-Validation (LOOCV) applied to 84 skin samples of Indo-Pacific bottlenose dolphins (*Tursiops* *aduncus*) for which age was known with an accuracy of ± 2 years and an age range between (mean ± SD = 15.2 ± 8.2). Ages were calculated using a) the species-specific clock developed in this study, b) the skin clock developed for *T. truncatus* (Robeck et al., 2021a), c) the skin and blood clock developed for *T. truncatus* (Robeck et al., 2021a), d) the skin clock for multi-species odontocetes (Robeck et al., 2021b), and e) the skin and blood clock for multi-species odontocetes (Robeck et al., 2021b). Regression lines are shown in blue (*T.* *aduncus* clock), orange (*T. truncatus* clocks) and red (multi-species odontocete clocks), dotted diagonal indicates a perfect correlation (y = x), points represent individual animals. Pearson correlation (r) and median absolute error (MAE) are given for each model.
